# Amygdala Atrophy and Its Functional Disconnection with the Cortico-Striatal-Pallidal-Thalamic Circuit in Major Depressive Disorder in Females

**DOI:** 10.1371/journal.pone.0168239

**Published:** 2017-01-20

**Authors:** Jie Yang, Yingying Yin, Connie Svob, Jun Long, Xiaofu He, Yuqun Zhang, Zhi Xu, Lei Li, Jie Liu, Jian Dong, Zuping Zhang, Zhishun Wang, Yonggui Yuan

**Affiliations:** 1 School of Information Science and Engineering, Central South University, Changsha, Hunan, China; 2 Department of Psychosomatics and Psychiatry, ZhongDa Hospital, School of Medicine, Southeast University, Nanjing, PR China; 3 Institute of Psychosomatics, Medical School of Southeast University, Nanjing, PR China; 4 Department of Psychiatry, Columbia University College of Physicians and Surgeons, New York, NY, United States of America; University of Texas at Austin, UNITED STATES

## Abstract

**Background:**

Major depressive disorder (MDD) is approximately twice as common in females than males. Furthermore, female patients with MDD tend to manifest comorbid anxiety. Few studies have explored the potential anatomical and functional brain changes associated with MDD in females. Therefore, the purpose of the present study was to investigate the anatomical and functional changes underlying MDD in females, especially within the context of comorbid anxiety.

**Methods:**

In this study, we recruited antidepressant-free females with MDD (*N* = 35) and healthy female controls (HC; *N* = 23). The severity of depression and anxiety were evaluated by the Hamilton Depression Rating Scale (HAM-D) and the Hamilton Anxiety Rating Scale (HAM-A), respectively. Structural and resting-state functional images were acquired on a Siemens 3.0 Tesla scanner. We compared the structural volumetric differences between patients and HC with voxel-based morphometry (VBM) analyses. Seed-based voxel-wise correlative analyses were used to identify abnormal functional connectivity. Regions with structural deficits showed a significant correlation between gray matter (GM) volume and clinical variables that were selected as seeds. Furthermore, voxel-wise functional connectivity analyses were applied to identify the abnormal connectivity relevant to seed in the MDD group.

**Results:**

Decreased GM volume in patients was observed in the insula, putamen, amygdala, lingual gyrus, and cerebellum. The right amygdala was selected as a seed to perform connectivity analyses, since its GM volume exhibited a significant correlation with the clinical anxiety scores. We detected regions with disrupted connectivity relevant to seed primarily within the cortico-striatal-pallidal-thalamic circuit.

**Conclusions:**

Amygdaloid atrophy, as well as decreased functional connectivity between the amygdala and the cortico-striatal-pallidal-thalamic circuit, appears to play a role in female MDD, especially in relation to comorbid anxiety.

## Introduction

Epidemiological studies have consistently demonstrated gender differences in prevalence rates of unipolar major depressive disorder (MDD), with females being twice as likely than males to develop it [1, 2]. The higher prevalence rates is believed to be a consequence of biological vulnerabilities and stressful environmental factors unique to females [3]. These include, but are not limited to, female-specific sex hormones, predisposing vulnerabilities to anxiety, and prolonged fixations on stressful experiences [3]. As a result, females differ from males with respect to symptomatology. Females with MDD, in contrast to males with MDD, are more likely to show anxiety, somatization, psychomotor retardation, and changes in sleep patterns [3]. Whereas much is known about the clinical aspects of MDD in females, there remains a need to identify the neural basis of MDD in order to help patients to lead asymptomatic lives.

Recently, researchers have investigated structural and functional neurological differences between female patients with MDD and healthy controls (HC) to better understand the neurobiological mechanisms of MDD [4, 5]. For example, studies of depression-associated brain abnormalities have highlighted the role of the cortico-striatal-pallidal-thalamic circuit in the pathophysiology of depression [6]. Further, given that disengaging from negative stimuli is often a clinical observation in depression, numerous neuroimaging studies have consistently reported MDD patients displaying attenuated activity during the exposure of negative stimuli in the prefrontal regions, such as the dorsolateral frontal cortex (DLPFC) and ventrolateral frontal cortex (VLPFC), which play vital roles in attention switching [7, 8]. As anhedonia is a notable symptom of MDD (the inability to experience pleasure and non-motivation to engage in activities usually related to pleasure) [9], it is not surprising that the striatal system is implicated in MDD. Morphological studies have reported decreased GM volume of the caudate in MDD patients, as well as increased activity of the striatal system in response to negative emotions [10, 11]. In this context, pallidus and thalamus have not received much attention. However, a post-mortem study has demonstrated that there were significantly more neurons in the mediodorsal (37%) and anteroventral/anteromedial (26%) thalamic nuclei in subjects with MDD relative to the non-psychiatric comparison group [12]. Lastly, the limbic system has received special attention in empirical work for its role in emotion perception and encoding, social salience of environmental stimuli, anxiety and fear conditioning, and motivational forces in behavior. Taken together, an abundance of studies on depression have documented structure, functional activity, and functional connectivity abnormalities within the limbic system [13–15].

More recently, resting-state functional connectivity analyses have been used to capture the interactive level of neural systems when no task is being performed [16, 17]. Seed-based analyses are frequently applied to resting-state data. The seed-based method is a hypothesis-driven approach wherein a seed region is selected as a reference, and the temporal correlations between the seed region and other brain regions are calculated to identify a set of plausible functional connectivity alterations in neuropsychiatric disorders [18]. Given that the amygdala is commonly identified as the elementary structure related to emotional evaluation, it has been frequently localized as the reference region in neuropsychiatric disorders studies. Ramasubbu et al selected the amygdala as a seed to investigate altered intrinsic connectivity in MDD patients [19]. Reduced functional connectivity relevant to the amygdala has been observed in regions including the insula, striatal system, and VLPFC. In addition to the amygdala, regions such as the hippocampus, insula, putamen, and anterior cingulate cortex (ACC) have also been localized as reference seeds in previous studies of depression. For example, Lui et al selected thirteen regions of interest and documented associations between these regions and mood regulation, specifically identifying the disrupted functional connectivity between refractory and nonrefractory depression [20].

To identify brain abnormalities of depression in a systemic manner, we combined the voxel-based morphometry (VBM) and resting-state functional connectivity approach to investigate structural alterations and potentially disrupted functional connectivity, respectively. According to evidence from previous neuroimaging studies, we hypothesized that the structural alterations and disrupted connectivity of depression would be mainly observed in the cortico-striatal-pallidal-thalamic circuit. Additionally, given that female patients with MDD tends to show increased anxiety, including higher rates of comorbid anxiety disorders [3], we further hypothesized that the limbic system (e.g., amygdala, hippocampus) would be implicated in our findings.

## Materials and Method

All study procedures were approved by the medical Ethics committee of ZhongDa Hospital, Southeast University. Prior to obtaining consent, the capacity to provide informed consent for all potential participants was ascertained by two licensed psychiatrists. After explaining the study procedures, informed written consent was obtained from all participants. All study procedures were conducted in strict accordance with the Declaration of Helsinki.

### Study sample

A total of 35 antidepressant-free females with MDD (aged 44.54 ± 11.14 years) from the Department of Psychosomatics and Psychiatry, ZhongDa Hospital, Southeast University and 23 matched HC (aged 39.09 ± 14.35 years) were recruited between March 2012 and December 2014 via community advertisements. Participants were carefully screened in a semi-structured interview by two trained senior psychiatrists.

Participants in the MDD group were confirmed to meet the DSM-IV criteria for MDD [21]. The diagnostic procedure included history from the patients and their families; medical, neurological, and psychiatric examinations were also carefully performed. The severity of depressive and anxiety symptoms were assessed with the 24-item Hamilton Depression Rating Scale (HAM-D) and the Hamilton Anxiety Rating Scale (HAM-A), respectively [22, 23]. Female patients were excluded if they met of the following criteria: (1) they were less than 18 years old or greater than 60 years old; (2) another major psychiatric illness was present; (3) they reported current or long-term smoking, alcohol, or drug dependence; (4) serious physical ailments, primary neurological illness, organic brain disease (e.g., former stroke, cerebral vascular malformations, epilepsy), or former brain injury; (5) endocrine disorders (e.g., hypertension, diabetes, thyroid dysfunction); or (6) received antidepressant treatment or psychiatric therapy within the 6 months prior to their enrollment.

HC were physically healthy, had no history of severe illness, and no first degree relative with a history of psychiatric illness. Clinical assessments for the control group were conducted by an experienced psychiatrist in order to verify inclusion and exclusion criteria. Eligible participants were required to have a HAM-D score less than 8 points and to have met the exclusion criteria (1) and (3). Further, all participants were right-handed and were required to have no contraindications for MRI studies.

### Data acquisition

Imaging data were acquired on a Siemens 3.0 Tesla scanner with a 12-channel head coil. High-resolution 3-dimensional T1-weighted 3D scans were recorded in a magnetization prepared rapid gradient echo (MPRAGE) sequence (TR = 1900ms; TE = 2.48 ms; FA = 90°; acquisition matrix = 256 × 256; FOV = 250 × 250 mm^2^). Whole brain resting-state fMRI data were acquired using a gradient-recalled echo-planar imaging pulse sequence (TR = 2000 ms; TE = 25ms; FA = 90°; acquisition matrix = 64×64; FOV = 240×240 mm^2^; Total volumes = 240). During the 8-mintue resting-state fMRI scan, participants were instructed to lay still in the scanner, keep their eyes open, and refrain from falling asleep.

### Voxel-based morphometry analysis

VBM analyses were carried out using Statistical Parametric Mapping software (SPM8: Wellcome Trust Centre for Neuroimaging, London, UK) run on Matlab 2014a (Math-Works, Natick, MA, USA). First, MR images were segmented into gray matter (GM), white matter (WM), and cerebrospinal fluid (CSF) using the standard unified segmentation module in SPM8 [24]. Second, study-specific GM templates were generated from the entire image dataset using the Exponentiated Lie algebra (DARTEL) method, an improved VBM method for greater accuracy in inter-subject brain registration. Third, after an initial affine registration of the GM DARTEL templates to the corresponding tissue probability maps in the Montreal Neurological Institute (MNI) space, non-linear warping of GM images were conducted to match the corresponding MNI space GM DARTEL templates. Fourth, images were modulated to ensure that relative volumes of GM were preserved following the spatial normalization procedure. Lastly, the modulated, normalized GM images (voxel size 1.5 × 1.5 × 1.5 mm^3^) were smoothed with an 8-mm full-width at half-maximum Gaussian kernel.

We compared regional GM volume across the two groups (i.e., female patients with MDD vs female HC) with a two sample t-test after controlling for age. Statistical parametric maps were generated after multiple comparison analyses (AlphaSim, AFNI, signal voxel threshold of p < 0.005 and cluster size >396). Female patients with MDD were prone to increased anxiety, including higher rates of comorbid anxiety disorders. Consequently, we conducted Pearson correlations between (1) the GM volume of regions with structural deficits, and (2) symptoms of anxiety as measured by the HAM-A summary score to identify the relationship between structural abnormalities and clinical symptoms in female with MDD.

### Resting-state functional connectivity analysis

Connectivity analyses were performed using SPM8 software and the CONN-fMRI Functional toolbox [25]. Briefly, the imaging preprocessing steps included slice-time corrections, realignment, coregistration, normalization into the Montreal Neurological Institute (MNI) space, resampling at 2 mm^3^, and spatial smoothing with a Gaussian kernel of 8 mm^3^ full-width at half maximum. White matter, cerebrospinal fluid (CSF), and motion-relevant parameters were taken as confounds, following the component-based noise-correction method to minimize non-neural influences on functional MRI (fMRI) signals. The whole brain BOLD signal was excluded as a regressor to eliminate erroneous anti-correlations [26]. The residual time-series were temporally bandpass filtered (0.01–0.08 Hz) to reduce the effect of low and high frequency physiological noise [27]. Then, Pearson correlations were calculated between the time course of seed regions and the time course of all other voxels. The resultant correlation coefficients were converted to normally distributed scores using the Fisher z transformation.

Two-sample t-tests were conducted to compare the functional connectivity between the two groups (i.e., female patients with MDD vs female HC), controlling for age. Statistical parametric maps were generated after multiple comparison analysis (AlphaSim, AFNI, signal voxel threshold of *p* < 0.01 and cluster size >251). Similar to our VBM analysis, the mean Z-values of functional connectivity were retrieved from regions with abnormal functional connectivity and were used to compute Pearson correlations with anxiety symptoms as measured by the HAM-A summary score in the females with MDD group.

## Results

### Demographic characteristics and clinical symptoms

Demographic and clinical characteristics of female patients with MDD and HC are presented in Table 1. There were no significant differences between the two groups with respect to age and years of education. Mean duration of illness was 5.82 months (SD = 2.86) for female MDD.

**Table 1:** Demographic and neuropsychological data.

Items	Female MDD (N = 35)	Healthy Females (N = 23)	T value	*P* value
Age (Years)	44.54±11.15	39.09±14.35	1.24	0.11*
Education (Years)	10.4±3.85	12.18±4.58	0.92	0.18*
HAM-D	28.29±7.99	N/A	N/A	N/A
HAM-A	20.17±7.17	N/A	N/A	N/A
Age of onset(years)	41.25±10.07	N/A	N/A	N/A
Total duration(months)	32.17±45.02	N/A	N/A	N/A
Current duration(months)	5.83±8.22	N/A	N/A	N/A
Frequency(times)	4.46±10.83	N/A	N/A	N/A
Family history(N/Y)	30/2	N/A	N/A	N/A

Note

* *P* value was calculated by Independent two-sample t-test.

Abbreviation: MDD, major depressive disorder; N, number; HAM-D, Hamilton Depression Rating Scale; HAM-A, Hamilton Anxiety Rating Scale; N/Y, No/Yes; N/A, not available.

### Voxel-based morphometry analysis

**Fig 1:**
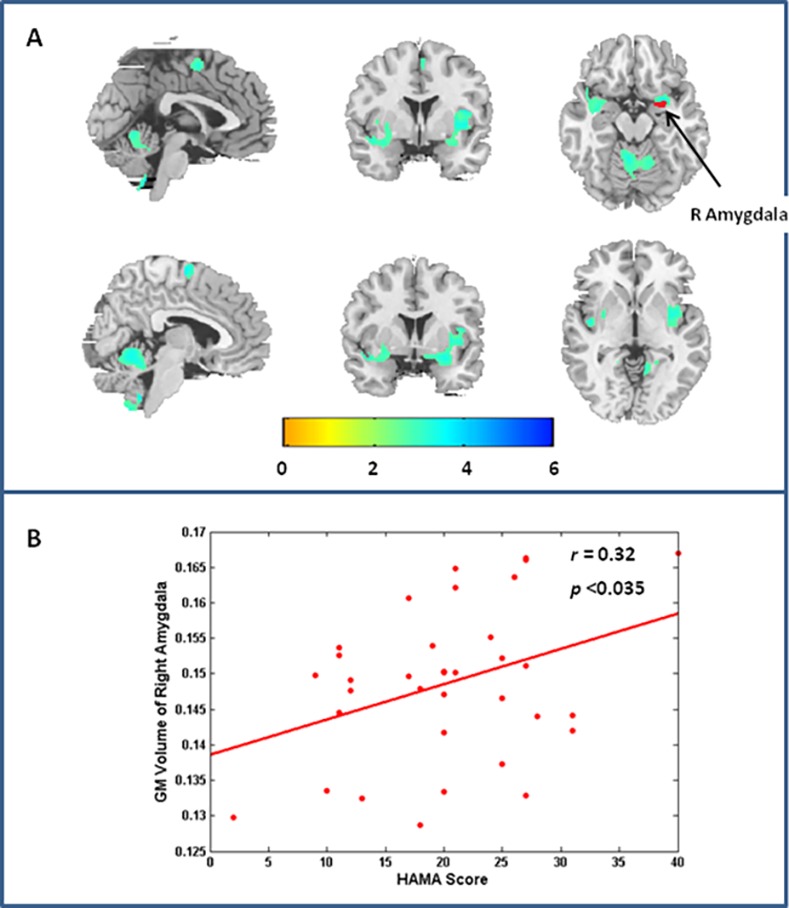
Regions showing significantly altered GM volume in females with MDD compared to HC. Panel A shows the regions representing significantly decreased GM volume in females with MDD compared to HC, including bilateral insula, bilateral amygdala, right hippocampus, bilateral parahippocampus gyrus, bilateral putamen, bilateral lingual gyrus, cerebellum, and caudal middle frontal regions. In contrast, no significant increase in GM volume was detected among females with MDD compared to HC. The significance level was set as single voxel threshold of *p*<0.005 and cluster size > 396 voxels, using AlphaSim correction. Panel B shows the right amygdala, with structural deficits, exhibits a significant correlation between GM volume and anxiety scores in females with MDD (r = 0.32, *p* = 0.035).

As Fig 1 and Table 2 present, female patients with MDD displayed decreased volume in the right amygdala, right parahippocampus gyrus (PHIPP), bilateral insula, bilateral putamen, left lingual gyrus (LING), cerebellum, and caudal middle-frontal region, when compared to the HC. Regions with structural deficits observed in our study were roughly symmetrical, but implicated the limbic system to a greater extent in the right hemisphere than in the left. In contrast, no significant increase in GM volume was found among female patients with MDD.

**Table 2:** The locations of regions showing significantly altered GM volume in females with MDD compared to HC.

Regions	MNI			Voxel Size	T value*
	x	y	z		
**Female MDD < Females HC**					
caudal middle frontal regions	-4.5	9	64.5	452	-3.66
Left Insula	-43.5	-7.5	-3	255	-3.36
Right Insula	39	0	1.5	539	-3.6
Left Putamen	-27	6	-9	123	-2.98
Right Putamen	33	0	1.5	580	-3.58
Right Amygdala	24	6	-15	91	-3.41
Right ParahippocampaGyrus	22.5	4.5	-19.5	62	-2.94
Left Lingual	-12	-51	-9	232	-3.86
Cerebellum	-52.5	6	-58.5	2343	-4.17
**Female MDD >Female HC**					
N/A					

*p<0.005, corrected for multiple comparisons using cluster extent thresholding method where the cluster size of 396 voxels was determined using Monte Carlo simulation (AlphaSim)

As female patients with MDD tended to display elevated anxiety traits, we conducted Pearson correlations between anxiety scores and the GM volume of regions showing structural deficits. As Fig 1 demonstrates, we detected that the right amygdala with structural deficits exhibited a significant correlation with anxiety scores in female patients with MDD (r = 0.32, *p*<0.035).

### Resting-state functional connectivity analysis

**Fig 2:**
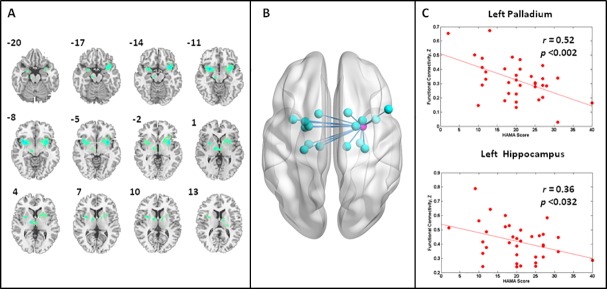
Regions showing significantly altered connectivity with the amygdala in females with MDD compared to HC. Panel A shows significantly altered connectivity with the amygdala in females with MDD compared to HC. Panel B shows the statistical maps rendered on a SurfTemplate by using the BrainNet Viewer (http://www.nitrc.org/projects/bnv); the magenta dot is the seed–the right amygdala; aquamarine dots indicate the right VLPFC, bilateral insula, bilateral parahippocampus, bilateral putamen, bilateral pallidus, and bilateral thalamus that displayed significantly decreased connectivity with the amygdala in females with MDD relative to HC. In contrast, no significant increased functional connectivity relevant to seed was detected among females with MDD compared to HC. The significance level was set as single voxel threshold of *p*<0.01 and cluster size > 251 voxels, using AlphaSim correction. Panel C shows significant correlations between connectivity and anxiety scores; the upper row displays the connectivity of the left pallidus, relevant to the right amygdala, significantly correlates with anxiety scores (r = 0.52, *p* = 0.0017); the bottom row displays the connectivity of the left hippocampus, relevant to the right amygdala, significantly correlates with anxiety scores (r = 0.36, *p* = 0.0314).

In following-up analysis on resting-state brain activity, the right amygdala was chosen as the seed region. Low frequency functional connectivity maps for the whole brain were generated for each group. As Fig 2 and Table 3 demonstrate, females with MDD displayed attenuated functional connectivity in the VLPFC, bilateral insula, and bilateral putamen compared with the HC. In contrast, no significant increased functional connectivity relevant to seed (i.e., right amygdala) was detected in females with MDD.

**Table 3:** The locations of the regions showing significantly altered connectivity with the amygdala in females with MDD compared to HC.

Regions	MNI			Cluster Size	T value*
	x	y	z		
**Female MDD < Females HC**					
Right VLPFC	24	10	22	75	-3.6
Left Insula	-36	14	-14	171	-4.01
Right Insula	38	12	-6	181	-4.26
Left Caudate	-10	-4	14	64	-2.84
Right Caudate	22	18	14	103	-3.13
Left Putamen	-30	6	-4	152	-4.28
Right Putamen	30	6	-10	612	-4.08
Left Pallidus	-14	8	2	53	-2.86
Right Pallidus	24	2	-6	83	-2.81
Left Hippocampus	-16	-16	-20	57	-3.09
Right Hippocampus	24	-4	-22	67	-3.15
Left Parahippocampa Gyrus	-16	-20	-22	87	-3.55
Right Parahippocampa Gyrus	18	-18	-22	159	-2.94
Left Thalamus	-2	-12	2	62	-3.10
Right Thalamus	14	-10	2	144	-3.87
**Female MDD > Female HC**					
N/A					

*p<0.01, corrected for multiple comparisons using cluster extent thresholding method where the cluster size of 251 voxels was determined using Monte Carlo simulation (AlphaSim)

Additionally, we observed aberrant connectivity of the left hippocampus (Peak MNI coordinate: X = -25; Y = -7; Z = -15; cluster size = 57; r = 0.36; *p*<0.032) and the left pallidus (Peak MNI coordinate: X = -18; Y = -6; Z = 8; cluster size = 53; r = 0.52; *p*<0.002) correlated significantly with anxiety scores (Fig 2).

## Discussion

The prevalence rates of MDD is one of the most consistent findings demonstrated in epidemiological studies of depression, with MDD being about twice as common in women as in men [1, 2]. However, the biological mechanism underlying MDD in females is still ambiguous, especially given its high prevalence of comorbid anxiety. There is growing interest in probing structural and functional patterns of brain abnormities related to MDD in females [28]. To our knowledge, this is the first study that combines VBM and resting-state functional connectivity to investigate brain abnormalities in females with MDD, compared to HC. In females with MDD, we observed structural deficits in the right amygdala, bilateral putamen, caudal middle-frontal region, LING, and the cerebellum. The right amygdala showed a significant correlation between decreased GM volume and anxiety scores. As such, we selected it as a seed region to conduct functional connectivity analyses. Regions with aberrant connectivity had a large overlap with regions that showed decreased GM volume; the overlapping regions were primarily constituents of the limbic-striatal circuit. The limbic-striatal circuit, together with regions that included the VLPFC and bilateral thalamus (i.e., regions that emerged specifically in the statistical map of aberrant connectivity), further corroborated previous findings that the cortico-striatal-pallidal-thalamic loop is strongly implicated in depression.

In support of our findings, previous neuroimaging studies have also demonstrated that females with MDD displayed significantly decreased GM volume in the amygdala, caudate, and insula [10, 29, 30]. A recent study combined thickness and VBM analyses to explore potential morphological abnormities of females with MDD and high-risk females [31]. Consistent with our results, the authors found that females with MDD exhibited reduced thickness in the caudal middle-frontal region compared to HC. The caudal part of the middle-frontal regions corresponds to the premotor region, which plays a pivotal role in task switching, especially in proactive behavioral switching [32]. The cerebellum, another region that manifested structural deficits in females with MDD, plays a vital role in motor coordination and motor behavior, as well as emotional processing. Several lines of evidence suggest that the cerebellum is involved in the pathology of depression, including structural abnormalities and irregular activation patterns during the resting-state, as well as dysfunction during cognitive and emotional tasks [33–35].

Moreover, convergent evidence from therapeutics, neuroimaging, and lesion studies suggests that amygdala disturbance is implicated in the pathophysiology of depressive illness [13, 36, 37]. Our finding of decreased GM volume in the amygdala of females with MDD further supports the critical role of the amygdala in the etiology of depression. Importantly, based on the initial structural deficit observed in the amygdala, we conducted an additional analysis of its resting-state co-activation with other functionally relevant regions by using the amygdala as a seed region. The purpose of this functional connectivity analysis was to test an amygdala-centered abnormal coupling pattern in females with MDD.

Similar to the amygdala, the hippocampus is also a critical component of the limbic system. It is involved in emotional memory, as well as the etiology and persistence of depressive symptoms, such as anxiety [38, 39]. We speculate that the attenuated connectivity between the hippocampus and the right amygdala in females with MDD, compared to HC, may be related to a deficit in the encoding and retrieval of emotional memories in females with MDD. This possibility is further corroborated by statistically significant correlations observed between connectivity strength and HAM-A scores.

Within the prefrontal lobe, we observed attenuated connectivity between the right VLPFC and the right amygdala in females with MDD (the same circuit believed to be involved in attention switching). It has been shown that, compared to HC, patients with depression tend to be attentionally-biased toward negatively-valenced stimuli and are less able to process positive information [40, 41]. Given the VLPFC’s role in stimulus selection and the amygdala’s role in emotion regulation, we conjecture that the difficulty patients with MDD have in disengaging from negative stimuli may be related to the attenuated activity we have observed.

Aberrant connectivity between the insula and the amygdala in patients with MDD has also been reported in neuroimaging studies that use seed-based or ICA methods [15, 42]. The insula, especially its left anterior part, appears to serve as an integrative hub and to be integral in the role of switching between brain networks. Conversely, the insula’s posterior (or, right part) has been implicated in the detection and interpretation of internal bodily states. From this, we can surmise that the perception and interpretation of internal bodily states (e.g., physiological discomfort such as increased heart rate) share common features with sensitivity to anxiety. The present findings suggest that the structural deficits in the insula, and the aberrant connectivity related to the amygdala, are centralized in the insula’s posterior (or, right) region and that these brain abnormalities may be associated with elevated sensitivity to anxiety (which is often observed in females with MDD).

It is also worth noting that we found decreased connectivity between striatal systems and the amygdala in females with MDD, compared with HC, which is consistent with previous studies on depression [19]. Animal models have also documented that the amygdala has widespread projections of several components of the striatal system, including the caudate and nucleus accumbens, as well as the putamen [43], which suggests that both the amygdala and the striatal system contribute to emotional and motivational behavior. Thus, we may suspect that the decoupling of the amygdala and striatal systems is associated with the anhedonia observed as a core symptom of MDD. Interestingly, we detected that aberrant connectivity of the left pallidus (relevant to the right amygdala) significantly correlated with symptoms of anxiety. A previous study found that the neural activity of the right amygdala significantly correlated with anhedonia in patients with MDD in response to positively-valenced stimuli [44]. Indeed, it seems intuitively true that high levels of anxiety would be incompatible with pleasurable experiences. Additionally, given our finding that the striatal system and amygdala both manifested structural deficits in females with MDD, it is reasonable to suspect that the structural deficits provide a potential mechanistic account for projection lesions and, hence, lead to attenuated coupling in amygdalostriatal circuits.

The thalamus, not just a relay station, but also a highly interactive structure that provides selection and transformation to different inputs, has recently been subjected to intense scrutiny in depression [12, 30, 45]. Our results of attenuated connectivity between the right amygdala with the thalamus in females with MDD, relative to HC, corroborates the convergent conclusion supported by many researchers that the direct projections from the amygdala to the dorsal hypothalamic are involved in symptoms of fear and anxiety [46, 47]. Furthermore, these symptoms were generally more pronounced in females with MDD [3, 48]. Another dimension to consider of the role of the thalamus in depression is its functional implications in pain perception and alertness. That is, somatization and sleep-related symptoms commonly observed in MDD [3] may stem from the hypoconnectivity of the right thalamus observed in females with MDD [49, 50].

Taken together, our findings shed light on neural mechanisms underlying the higher incidence rates of MDD in females as compared to males. The present study, benefiting from its unique focus on a female population, has outlined a set of brain regions with alterations at both anatomical and functional levels in females with MDD. It has been long suspected that certain brain abnormalities are responsible for the female predisposition for MDD. Hastings et al have demonstrated that females with MDD relative to males with MDD, displayed decreased GM volume in the amygdala [51]. Meanwhile, a recent study of sex-related patterns of morphological abnormalities has demonstrated that females with MDD had altered GM volume in the frontal-limbic circuits, suggesting that the frontal-limbic circuits subserve emotions that are associated with increased depression or anxiety risk in females [30]. Empirical evidence has pointed to links between sex and clinical manifestation of MDD: females with MDD are prone to display anxiety and comorbid anxiety disorder, whereas males with MDD are more likely to display impulsiveness [3, 48]. As such, sex is a factor worthy of further consideration. Our study has added new insight into the pathological mechanism of MDD in females. In contrast to most other published work on this subject, we take advantage of two modalities, that is, the combined analysis of anatomical and resting-state imaging. By doing so, we have been able to help identify a female MDD neural bases in brain anatomy and activity.

## Limitations

There are several limitations to our study that should be noted. First, the small sample size may have limited our ability to detect statistical group differences. For example, we did not detect the disrupted connectivity of the ACC relevant to seed, which has consistently appeared in other functional connectivity studies. Second, some potential confounding factors, such as physiological noise (respiratory and cardiac rhythms) and the arousal level of participants during resting state scans were not controlled for in our analyses. Third, we used the AlphaSim correction for multiple comparisons, although options (e.g., FDR, FWE) are also available and could be used. Fourth, our conclusions were based on an all-female cohort. Any attempt to generalize our findings to a male population should be made with caution.

## Conclusions

Our study applied VBM and resting-state functional connectivity analyses to identify the brain abnormities of females with MDD at a systemic level. The study suggests that amygdaloid atrophy, as well as decreased functional connectivity between the amygdala and the cortico-striatal-pallidal-thalamic circuit, play an important role in female MDD, especially in relation to comorbid anxiety. Our study has added new insight into the female-specific pathological mechanism of MDD, and has emphasized the role of the amygdala in the pathophysiology of MDD in females.

## Supporting Information

S1 Dataset Functional connectivity data from all patients and healthy controls. (ZIP)

S2 Dataset T1 structure data (13 patients). (ZIP)

S3 Dataset T1 structure data (9 patients and 4 healthy controls). (ZIP)

S4 Dataset T1 structure data (13 healthy controls). (ZIP)

S5 Dataset T1 structure data (13 healthy controls). (ZIP)

S6 Dataset T1 structure data (6 healthy controls). (ZIP)

S7 Dataset Statistical analysis results. (ZIP)
